# Oxidized phospholipids stimulate production of stem cell factor via NRF2-dependent mechanisms

**DOI:** 10.1007/s10456-017-9590-5

**Published:** 2018-01-12

**Authors:** Taras Afonyushkin, Olga V. Oskolkova, Valery N. Bochkov

**Affiliations:** 1 CeMM Research Center for Molecular Medicine of the Austrian Academy of Sciences, Lazarettgasse 14, AKH BT 25-3, 1090 Vienna, Austria; 2 Department of Pharmaceutical Chemistry, Institute of Pharmaceutical Sciences, University of Graz, Humboldtstrasse 46/III, 8010 Graz, Austria; 3 Department of Vascular Biology and Thrombosis Research, Center for Physiology and Pharmacology, Medical University of Vienna, Schwarzspanierstrasse 17, 1090 Vienna, Austria

**Keywords:** Oxidized phospholipids, SCF, c-Kit, NRF2, Electrophilic stress response, Atherosclerosis

## Abstract

Receptor tyrosine kinase c-Kit and its ligand stem cell factor (SCF) regulate resident vascular wall cells and recruit circulating progenitors. We tested whether SCF may be induced by oxidized palmitoyl-arachidonoyl-phosphatidylcholine (OxPAPC) known to accumulate in atherosclerotic vessels. Gene expression analysis demonstrated OxPAPC-induced upregulation of SCF mRNA and protein in different types of endothelial cells (ECs). Elevated levels of SCF mRNA were observed in aortas of ApoE^−/−^ knockout mice. ECs produced biologically active SCF because conditioned medium from OxPAPC-treated cells stimulated activation (phosphorylation) of c-Kit in naïve ECs. Induction of SCF by OxPAPC was inhibited by knocking down transcription factor NRF2. Inhibition or stimulation of NRF2 by pharmacological or molecular tools induced corresponding changes in SCF expression. Finally, we observed decreased levels of SCF mRNA in aortas of NRF2 knockout mice. We characterize OxPLs as a novel pathology-associated stimulus inducing expression of SCF in endothelial cells. Furthermore, our data point to transcription factor NRF2 as a major mediator of OxPL-induced upregulation of SCF. This mechanism may represent one of the facets of pleiotropic action of NRF2 in vascular wall.

**Electronic supplementary material:**

The online version of this article (10.1007/s10456-017-9590-5) contains supplementary material, which is available to authorized users.

## Introduction

Stem cell factor (SCF, KIT ligand, steel factor) is a growth factor activating receptor tyrosine kinase c-Kit, which is structurally related to the platelet-derived growth factor (PDGF) receptor and is widely recognized for its role in stem cell biology [1]. SCF produced in bone marrow by endothelial and perivascular stromal cells is a major player in forming a niche for c-Kit-positive hematopoietic stem cells [2]. Furthermore, SCF and c-Kit are important for differentiation and function of mast cells [3] and demonstrate multiple other biological activities. In particular, rapidly accumulating data point to the role of SCF/c-Kit in regulation of vascular wall homeostasis, which is however less investigated as compared to other effects of SCF and c-Kit.

Major vascular wall cells such as endothelial cells (EC) and vascular smooth muscle cells (VSMC) are both producers and targets of SCF [4–8]. Treatment of ECs with SCF stimulates pro-angiogenic reactions including survival and migration of ECs, as well as formation of endothelial capillary tubes [9]. In vivo data show that c-Kit deficiency inhibits proliferation of ECs and suppresses angiogenesis [10]. Furthermore, SCF and c-Kit protect VSMCs from apoptosis [11] and regulate contractile phenotype of these cells [5]. SCF and c-Kit play a role in vascular disease and repair as suggested by enhanced expression of c-Kit by resident cells, as well as recruitment of circulating c-Kit-positive cells in various vascular pathologies including atherosclerosis [12, 13], in-stent restenosis [14], restenosis of artificial vessel grafts [15], aortic aneurysms [16], idiopathic pulmonary arterial hypertension [17] and aging [18]. Altogether, available data support the notion that SCF and c-Kit play important and multifaceted roles in vascular biology and pathology thus justifying research into the factors and mechanisms regulating expression and function of these proteins.

Accumulation of oxidized low-density lipoprotein (LDL) within arterial wall is a hallmark of atherosclerosis. Phospholipids within LDL particles are prone to oxidation due to the high contents of polyunsaturated fatty acids. Oxidized phospholipids (OxPLs) are generated by enzymatic or non-enzymatic oxidation of esterified fatty acids and demonstrate a variety of activities in vitro and in vivo [19, 20]. The relevance of OxPLs to vascular pathology is illustrated by their ability to stimulate monocyte–endothelial interactions, inhibit endothelium-dependent relaxation of vessels, promote formation of foam cells, induce phenotypic modulation and migration of VSMCs, enhance thrombogenic activity of ECs and platelets and stimulate angiogenesis [21]. These effects are mediated by multiple signaling pathways including cellular stress reactions called unfolded protein response (UPR) and electrophilic stress response (ESR). The major transcriptional factors mediating UPR and ESR in OxPL-treated ECs are ATF4 and NRF2, respectively [22, 23]. OxPLs are known to accumulate in human and animal atherosclerotic vessels at high concentrations comparable to those inducing biological effects in vitro [24]. In this work, we asked a question whether OxPLs can stimulate production of SCF. Our data show that phospholipid oxidation products increase expression of SCF by endothelial and monocytic cells and that transcription factor NRF2 plays an important role in OxPL-induced upregulation of SCF.

## Results

### OxPAPC upregulates SCF mRNA and protein in endothelial cells and a monocytic cell line

**Fig. 1:**
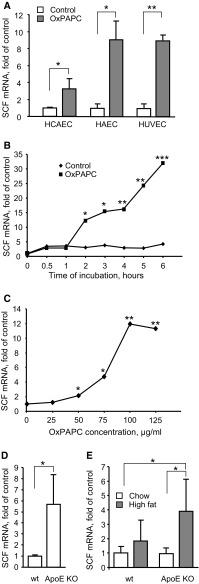
SCF mRNA is induced in endothelial and monocytic cells by OxPLs and PGA2 in a time- and concentration-dependent manner **a** Endothelial cells were stimulated with OxPAPC (100 µg/ml, 6 h). Total RNA was extracted using Trizol reagent and analyzed by microarray hybridization (HCAEC; three independent experiments and hybridizations) or qRT-PCR (HAEC, HUVEC; quadruplicate samples from one representative experiment out of three). Levels of SCF mRNA were normalized to β2-microglobulin mRNA. **b** HUVECs were stimulated with OxPAPC (100 µg/ml) for indicated time periods, followed by analysis of SCF mRNA. **c** HUVECs were treated with indicated concentrations of OxPAPC for 6 h. qRT-PCR was used for SCF mRNA quantification. **d**, **e** Levels of SCF mRNA were analyzed in aortas of aged (12 months old) wild type and ApoE^−*/*−^ mice (**d**) or in aortas of wild type and ApoE^−*/*−^ young animals fed for 8 weeks either with chow (6.5% fat) or high-fat diet (15% fat) (**e**). Total RNA was prepared from homogenized aortas using Trizol reagent. SCF mRNA expression was analyzed by qRT-PCR and normalized to β2-microglobulin mRNA levels

Treatment of ECs with OxPAPC is known to regulate expression of hundreds of genes in HAECs [25]. We applied microarray hybridization in order to analyze effects of OxPAPC on gene expression in another type of arterial ECs, i.e., HCAEC. One of physiologically important genes that were upregulated by OxPAPC in HCAECs was KIT ligand (*KITL*), more often referred to as stem cell factor (SCF). Elevation of SCF mRNA levels in HCAECs was observed in three independent experiments (Fig. 1a). Real-time PCR analysis confirmed upregulation of SCF mRNA in another type of aortic ECs (HAEC), as well as in venous ECs (HUVEC, Fig. 1a). Furthermore, OxPAPC upregulated SCF mRNA in the human monocytic cell line THP-1 (Suppl. Fig. 1). In addition to OxPAPC, SCF was upregulated in HUVECs by OxLDL and lipid peroxidation product such as electrophilic isoprostaglandin A_2_ (Suppl. Fig. 2).

Upregulation of SCF mRNA was time-dependent (Fig. 1b) and was observed within the range of OxPAPC concentrations known to be present in atheroma [24] (Fig. 1c). Furthermore, we observed statistically significant elevation of SCF mRNA in aortas from aged *ApoE* knockout mice (Fig. 1d), as well as in younger animals fed high-fat diet (Fig. 1e).

**Fig. 2:**
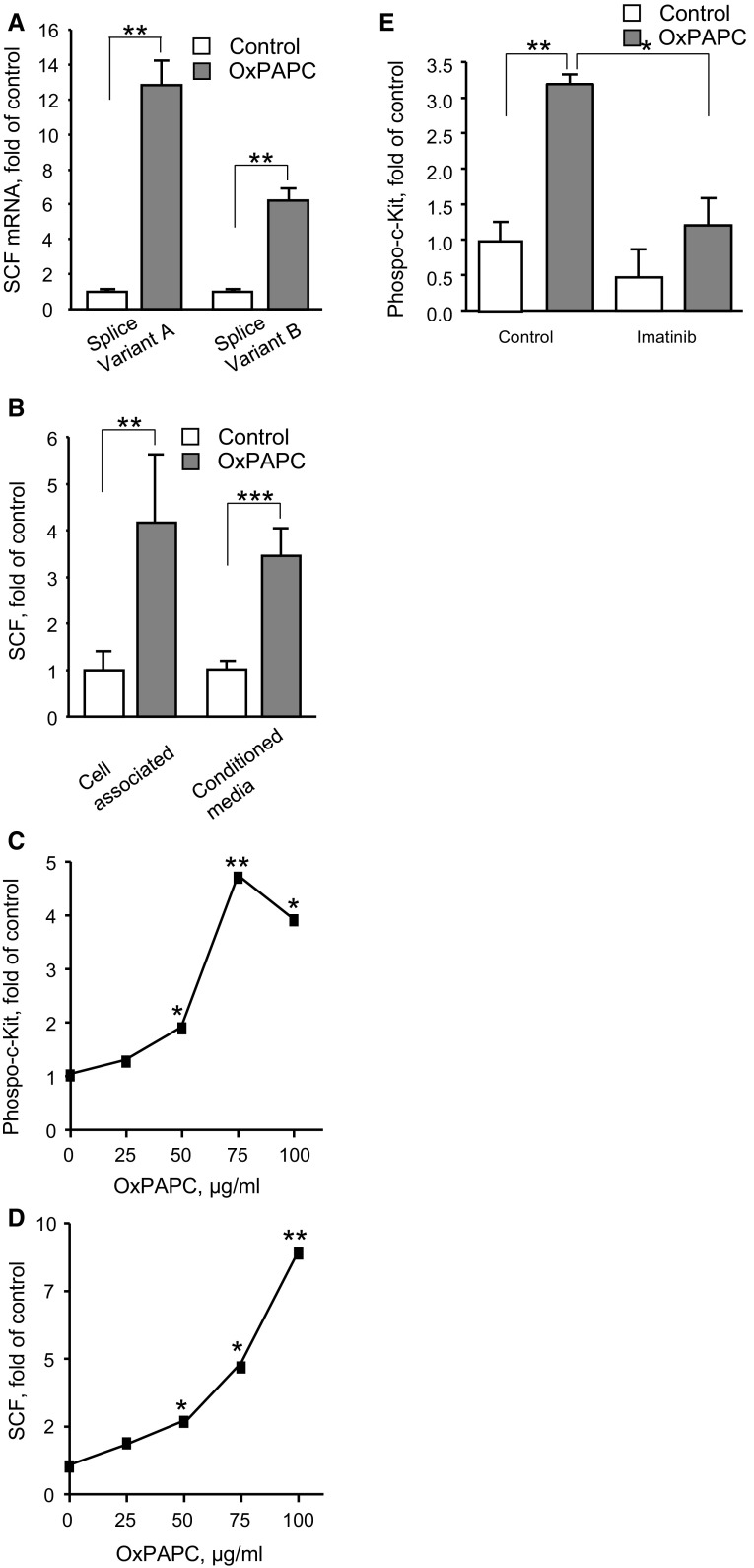
OxPAPC induces expression of cell-associated and secreted SCF protein and stimulates c-Kit phosphorylation. **a** Two forms of SCF mRNA were quantified in OxPAPC-stimulated HUVECs (100 µg/ml, 6 h) by qRT-PCR using primers selective for individual splice variants. **b** SCF protein was analyzed by ELISA in extracts of HUVECs or in conditioned media after 12 h of stimulation with 100 µg/ml OxPAPC. **c** Conditioned media produced by cells stimulated with indicated concentrations of OxPAPC stimulate phosphorylation of c-Kit receptor in HUVECs. **d** Panel presents ELISA data on the levels of SCF protein in conditioned media. **e** Pretreatment of cells with c-Kit tyrosine kinase inhibitor imatinib (30 min, 20 µM) attenuated c-Kit phosphorylation in HUVECs incubated with conditioned media from OxPAPC-stimulated cells

The analysis of SCF mRNA splice variants demonstrated the presence of two forms. One of them is known to encode both membrane and soluble variants of SCF, while another mainly produces the membrane form [26] (Fig. 2a). In a good agreement with this finding, Western blotting detected two anti-SCF positive bands corresponding to expected sizes of the two isoforms (Suppl. Fig. 3). Furthermore, SCF protein was detected both in conditioned medium from OxPAPC-treated cells and in cell lysates (Fig. 2b). Thus, we hypothesize that OxPAPC upregulated both soluble and membrane isoforms of SCF.

We further tested whether SCF produced by OxPAPC-treated cells was biologically active. To this end, HUVECs were stimulated by conditioned medium from OxPAPC-treated cells, followed by analysis of activation (autophosphorylation) of the SCF receptor, c-Kit. Conditioned medium induced phosphorylation of c-Kit (Fig. 2c). The phosphorylation developed within the same range of OxPAPC concentrations where it stimulated SCF secretion (Fig. 2d) and was inhibited by c-Kit inhibitor imatinib (Fig. 2e). These data suggest that OxPAPC stimulated HUVECs to produce biologically active SCF.

### Oxidized phospholipids upregulate SCF via the NRF2 pathway

**Fig. 3:**
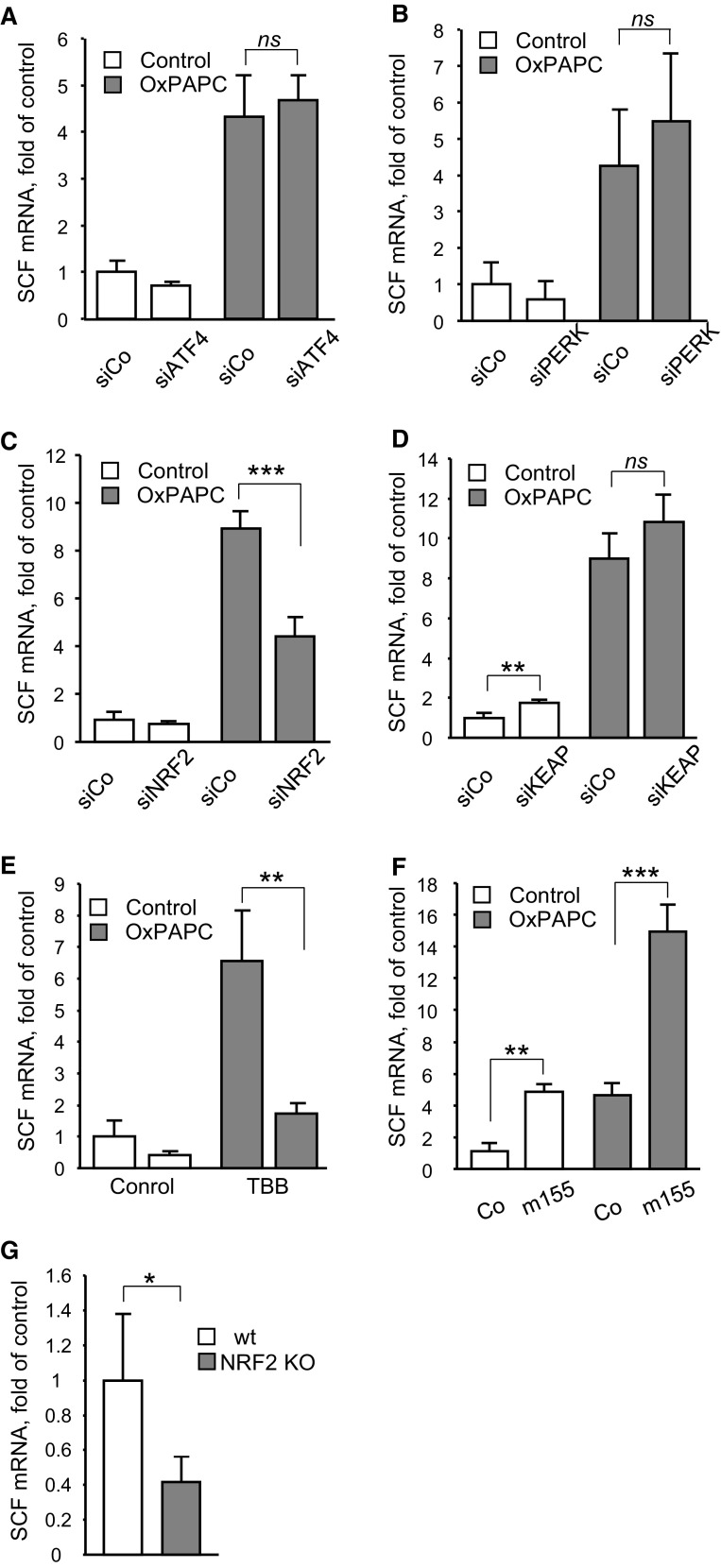
Induction of SCF by OxPAPC depends on the transcription factor NRF2. **a**, **b**, **c** and **d** HUVECs were transfected with siRNAs targeting ATF4 (**a**), PERK (**b**), NRF2 (**c**), or KEAP (**d**). Twenty-four hours after transfection cells were stimulated with OxPAPC (100 µg/ml, 6 h). Levels of SCF mRNA were analyzed by qRT-PCR in total RNA prepared using Trizol reagent and normalized to β2-microglobulin mRNA. **e** Protein kinase CK2 inhibitor TBB attenuates induction of SCF by OxPAPC. Cells were pretreated with TBB (20 µM, 30 min) and thereafter stimulated with OxPAPC (100 µg/ml, 6 h); SCF mRNA was quantified as described above. **f** miR-155 potentiates induction of SCF by OxPAPC. HUVECs were transfected with the RNA oligonucleotide mimicking miR-155 for 24 h and stimulated by OxPAPC (100 µg/ml, 6 h). Levels of SCF mRNA were quantified by qRT-PCR. **g** Steady-state levels of SCF mRNA are decreased in aortas of NRF2^−*/*−^ mice. Total RNA was prepared from aortas of 6 months old NRF2^−*/*−^ or wild-type mice and analyzed by qRT-PCR method. The levels of NRF2 mRNA were normalized to β2-microglobulin mRNA

OxPLs are known to activate electrophilic and unfolded protein stress responses (ESR and UPR, respectively), which play important role in regulation of gene expression by these lipids [22, 23]. The knockdown of two major components of UPR, i.e., PERK and ATF4, did not significantly influence induction of SCF by OxPAPC (Fig. 3a, b), although expression of VEGF, known as a target of PERK and ATF4 [23], was suppressed (Suppl. Fig. 4a, b). Furthermore, chemically different inducers of UPR such as tunicamycin, brefeldin and homocysteine upregulated VEGF but did not change SCF mRNA levels (Suppl. Fig. 5a, b). In contrast to the UPR pathway, transfection of cells with siRNA against the key transcriptional mediator of ESR, i.e., NRF2, resulted in significant inhibition of the OxPAPC-induced upregulation of SCF (Fig. 3c) and a reference NRF2 target gene OKL38 [27] (Suppl. Fig. 6a). The knockdown of the NRF2 inhibitor KEAP-1 did not enhance SCF or OKL38 induction by OxPAPC but reproducibly elevated basal mRNA levels of these genes (Fig. 3d and Suppl. Fig. 6b). These data allow hypothesizing that NRF2 is a signaling mediator of OxPAPC-induced upregulation of SCF.

Protein kinase CK2 plays an important role in control of ESR in HUVECs treated with OxPLs [28]. In agreement with these data, pretreatment of HUVECs with a specific CK2 inhibitor, TBB, significantly inhibited OxPAPC-induced upregulation of SCF (Fig. 3e) and a reference NRF2 target gene OKL38 (Suppl. Fig. 6c).

Levonen and colleagues have shown that microRNA miR-155 inactivated transcriptional regulator BACH1 [29]. In contrast to NRF2, which enhances transcription by binding at the antioxidant response element (ARE) of target genes, BACH1 acts as a repressor of ARE-dependent transcription [30]. We used oligonucleotides mimicking miR-155 as tools to check the importance of ARE-driven transcription in induction of SCF mRNA by OxPAPC. In agreement with the proposed mechanism, the oligonucleotide mimicking miR-155 elevated basal levels and enhanced OxPAPC-induced upregulation of SCF (Fig. 3f) and a reference NRF2 target gene OKL38 (Suppl. Fig. 6d).

Finally, we found that the levels of SCF mRNA in aortas of the NRF2^−/−^ mice were lower than in the wild-type animals (Fig. 3g), which further support our hypothesis about the involvement of NRF2 in upregulation of SCF under the conditions of oxidative stress and lipid oxidation.

## Discussion

Production of SCF by ECs is stimulated by inflammatory agonists and bacterial products [7, 12, 31]. In this work, we describe for the first time additional stimuli promoting expression of SCF, namely OxPLs and electrophilic prostanoids. These compounds accumulate in atherosclerotic vessels and are relevant to the initiation and progression of the disease [21, 32]. We observed upregulation of SCF in genetically hypercholesterolemic mice, which supports the notion that oxidized lipids upregulate SCF in atherosclerotic vessels. Induction of SCF by OxPLs potentially can have broad impact on atherogenesis due to ability of this growth factor to regulate viability, migration and differentiation of ECs and VSMCs, as well as recruit local and circulating progenitor and stem cells [5, 6, 9]. Furthermore, SCF is a key factor regulating differentiation of mast cells, as well as their recruitment into the arterial wall where mast cells play a role both in initiation and advanced stages of atherogenesis [33]. The importance of mast cells for atherogenesis was confirmed by decreased atherosclerosis in hyperlipidemic mice deficient in mast cells [34–36]. Altogether, available data suggest that upregulation of SCF can modulate several key mechanisms promoting development and progression of lesions.

OxPLs do not induce the major inflammatory pathway NFκB that is activated by inflammatory cytokines and bacterial products [19, 21], suggesting the involvement of additional mechanisms of SCF induction. In support of this possibility, we characterize NRF2-dependent transcription as a potential mechanism of SCF regulation by OxPLs. The involvement of NRF2 was shown using several approaches targeting different steps both upstream and downstream of NRF2 activation, including inhibition of protein kinase CK2 that is important for activation of electrophilic stress response by OxPLs [28], knockdown of key players in ESR such as KEAP-1 and NRF2, silencing of transcriptional repressor BACH1 by miR-155 and analysis of SCF expression in vivo in arteries of NRF2 knockout mice. All these data consistently support our hypothesis about the importance of the NRF2 pathway in upregulation of SCF by lipid oxidation products.

Previously, we have shown that the ESR pathway plays an important role in upregulation of VEGF in ECs treated with OxPLs [37]. Both VEGF and SCF protect endothelium from apoptosis and stimulate its regeneration by local mechanisms (migration, proliferation) as well as by mobilizing and attracting circulating progenitor cells. Thus, we hypothesize that the expression of VEGF and SCF in response to cellular stress induced by oxidized lipids represents a compensatory reaction aiming at the endothelial protection and repair. Our data show that these protective effects are mediated via the NRF2 pathway. However, prolonged secretion of VEGF and SCF potentially can attract monocytes, smooth muscle and mast cells and also stimulate plaque neovascularization thus leading to disease progression. Further in vivo studies are required in order to dissect specific role of OxPL-induced SCF in early and advanced stages of atherosclerosis.

## Materials and methods

### Materials, cell culture and lipid preparation

Tunicamycin, brefeldin, homocysteine, 4,5,6,7-tetrabromo-2-azabenzimidazole (TBB), imatinib and polyethylenimine (PEI) were obtained from Sigma-Aldrich. Human umbilical vein ECs (HUVECs), human carotid artery ECs (HCAECs) and human aortic ECS (HAECs) (all from Lonza) were grown at 37 °C in 5% CO_2_ in medium M199 containing 20% FCS, 1 U/ml heparin, ECGS growth supplement (Promocell), 2 mmol/l glutamine, 100 U/ml penicillin and 100 μg/ml streptomycin and used up to passage 5. 1-Palmitoyl-2-arachidonoyl-*sn*-glycero-3-phosphocholine (PAPC) (Avanti Polar Lipids) was oxidized by an exposure to air. Formation of OxPAPC was controlled by electrospray ionization-mass spectrometry and thin-layer chromatography [38]. For cell stimulation, the lipids were resuspended in medium M199 containing 2% fetal calf serum (FCS).

### cDNA synthesis and qRT-PCR analysis of RNA

ECs were treated either with indicated concentration of OxPAPC in medium M199 containing 2% serum or with control medium for 4 h. Trizol reagent (Invitrogen) was used for RNA isolation. Microarray (Affymetrix Gene Profiling Array) profiling of mRNAs and data analysis were done at the Medical University of Vienna Genomics Core Facility. GeneAmp RNA-PCR kit and Fast SYBR Green Master Mix were used for analysis of mRNA by qRT-PCR. All these reagents were purchased from Applied Biosystems. Sequences of primers are available upon request. StepOnePlus real-time PCR cycler (Applied Biosystems) was used for quantitative real-time PCR.

### Transfection with siRNA and miRNA mimic

ECs were transfected with 50 nM of either siRNA, miRNA mimic, or control oligonucleotide (all from Qiagen) in plain M199 medium for 4 h using polyethylenimine (PEI) reagent [39]. All stimulations were performed 24 h after transfection.

### ELISA and Western blotting

R&D Systems ELISA kit was used for measurements of the SCF level in cell lysates and conditioned medium. The degree of c-Kit autophosphorylation was analyzed by PathScan^®^ Phospho-c-Kit (Tyr719) sandwich ELISA kit. Signal detection was performed spectrophotometrically at 450 nm. For SCF analysis, protein samples were denatured in Laemmli buffer and separated in SDS-polyacrylamide gels with following electroblotting to PVDF membrane (Millipore). Protein blots were probed with anti-SCF antibodies (Cell Signaling Technology). Horseradish peroxidase conjugated IgG (GE Healtcare) and SuperSignal West Femto Substrate (Pierce) were used for detection of bound primary antibodies. Chemiluminescense was detected by using FluorChem HD2 imager (Alpha Innotech).

### Mice

Sixteen weeks old male C57BL/6 and ApoE^−/−^ mice (six animals per group) were fed either a standard laboratory chow diet (6.5% fat) or a proatherogenic diet containing 15% fat and 1.25% cholesterol (Sniff) for 8 weeks. Twelve months old C57BL/6 and ApoE^−/−^ mice hold on chow diet were used for comparison of mRNA levels in aged animals. Six months old C57BL/6 and NRF2^−/−^ mice hold on chow diet were used for comparison of the SCF mRNA levels. Total RNA was prepared from homogenized aortas using Trizol reagent (Invitrogen) and analyzed as described above. Experiments were approved by the Medical University of Vienna animal experimentation committee and the Austrian Ministry of Science.

### Statistical analysis

Two-tailed Student’s *t* test was used for analysis; *p* value less than 0.05 was considered significant. All data are represented as means ± standard deviations.

## Electronic supplementary material

Below is the link to the electronic supplementary material.

Supplementary material 1 (PPT 300 kb)

## Abbreviations

OxPAPC: Oxidized 1-palmitoyl-2-arachidonoyl-*sn*-glycero-3-phosphocholine
SCF: Stem cell factor
ESR: Electrophilic stress response
UPR: Unfolded protein response
ECs: Endothelial cells
OxPLs: Oxidized phospholipids
