# Supplementary material for: Oxidized phospholipids stimulate production of stem cell factor via NRF2-dependent mechanisms
Source: Angiogenesis. 2018 Jan 12;21(2):229–36. doi: 10.1007/s10456-017-9590-5 (PMC5878191; doi:10.1007/s10456-017-9590-5)
Supplement: Supplementary file 1 — Supplementary material 1 (PPT 300 kb) [file 10456_2017_9590_MOESM1_ESM.ppt]

## Slide 1
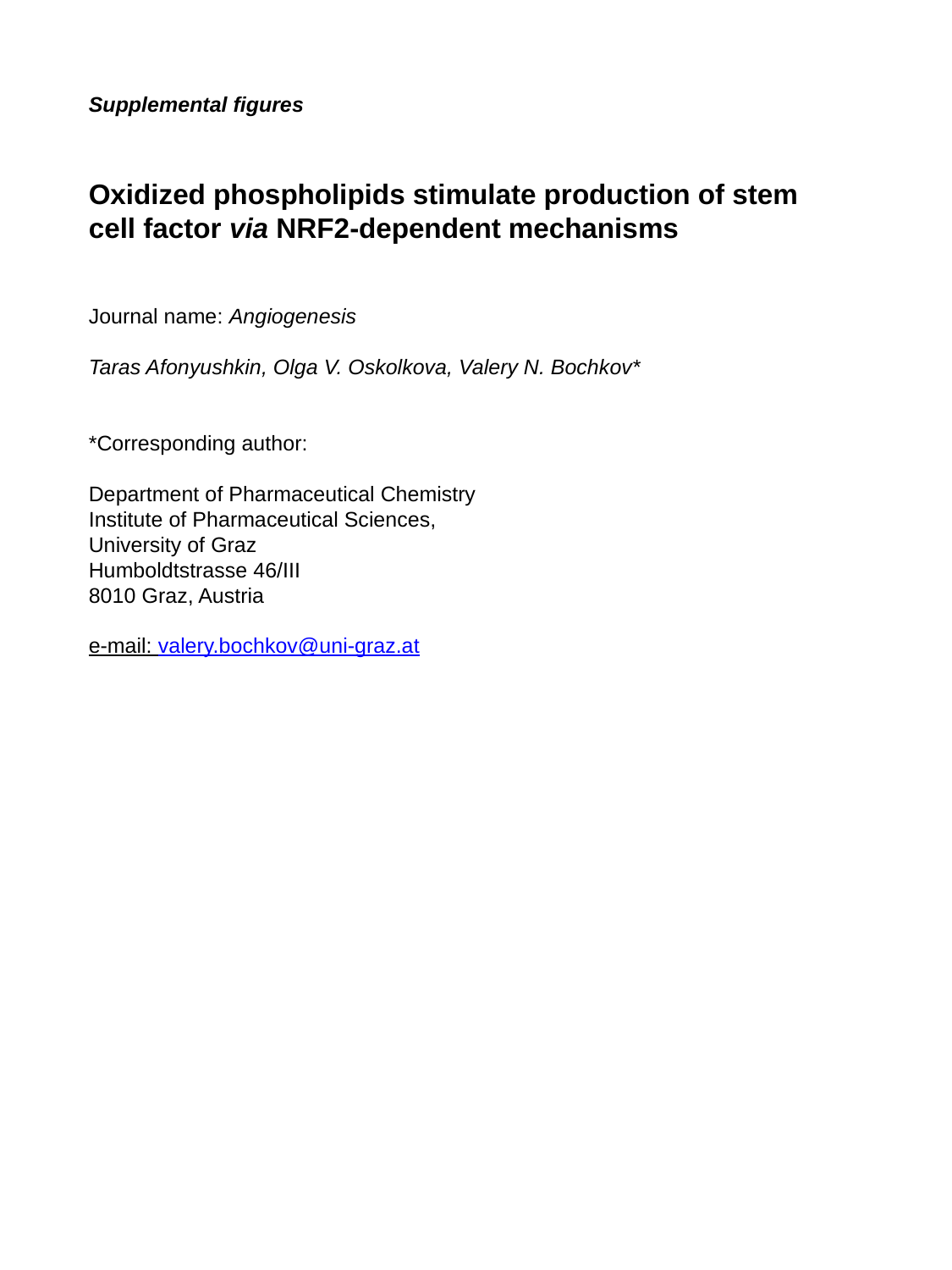

Supplemental figures
Oxidized phospholipids stimulate production of stem cell factor via NRF2-dependent mechanisms
Journal name: Angiogenesis
Taras Afonyushkin, Olga V. Oskolkova, Valery N. Bochkov*
*Corresponding author:
Department of Pharmaceutical Chemistry
Institute of Pharmaceutical Sciences,
University of Graz
Humboldtstrasse 46/III
8010 Graz, Austria
e-mail: valery.bochkov@uni-graz.at

## Slide 2
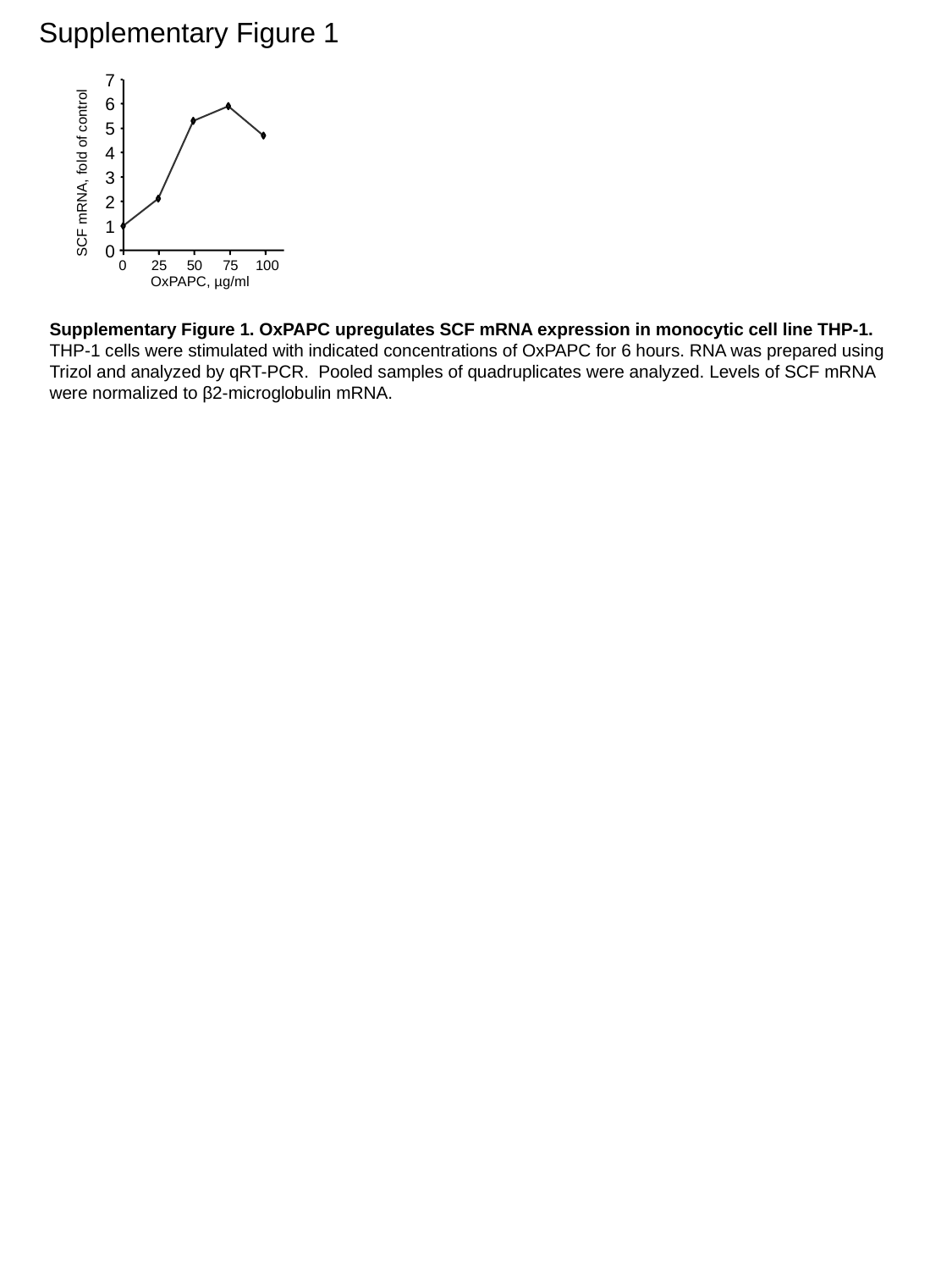

Supplementary Figure 1
7
0
25
50
75
100
OxPAPC, µg/ml
6
5
4
SCF mRNA, fold of control
3
2
1
0
Supplementary Figure 1. OxPAPC upregulates SCF mRNA expression in monocytic cell line THP-1.
THP-1 cells were stimulated with indicated concentrations of OxPAPC for 6 hours. RNA was prepared using Trizol and analyzed by qRT-PCR. Pooled samples of quadruplicates were analyzed. Levels of SCF mRNA were normalized to β2-microglobulin mRNA.

## Slide 3
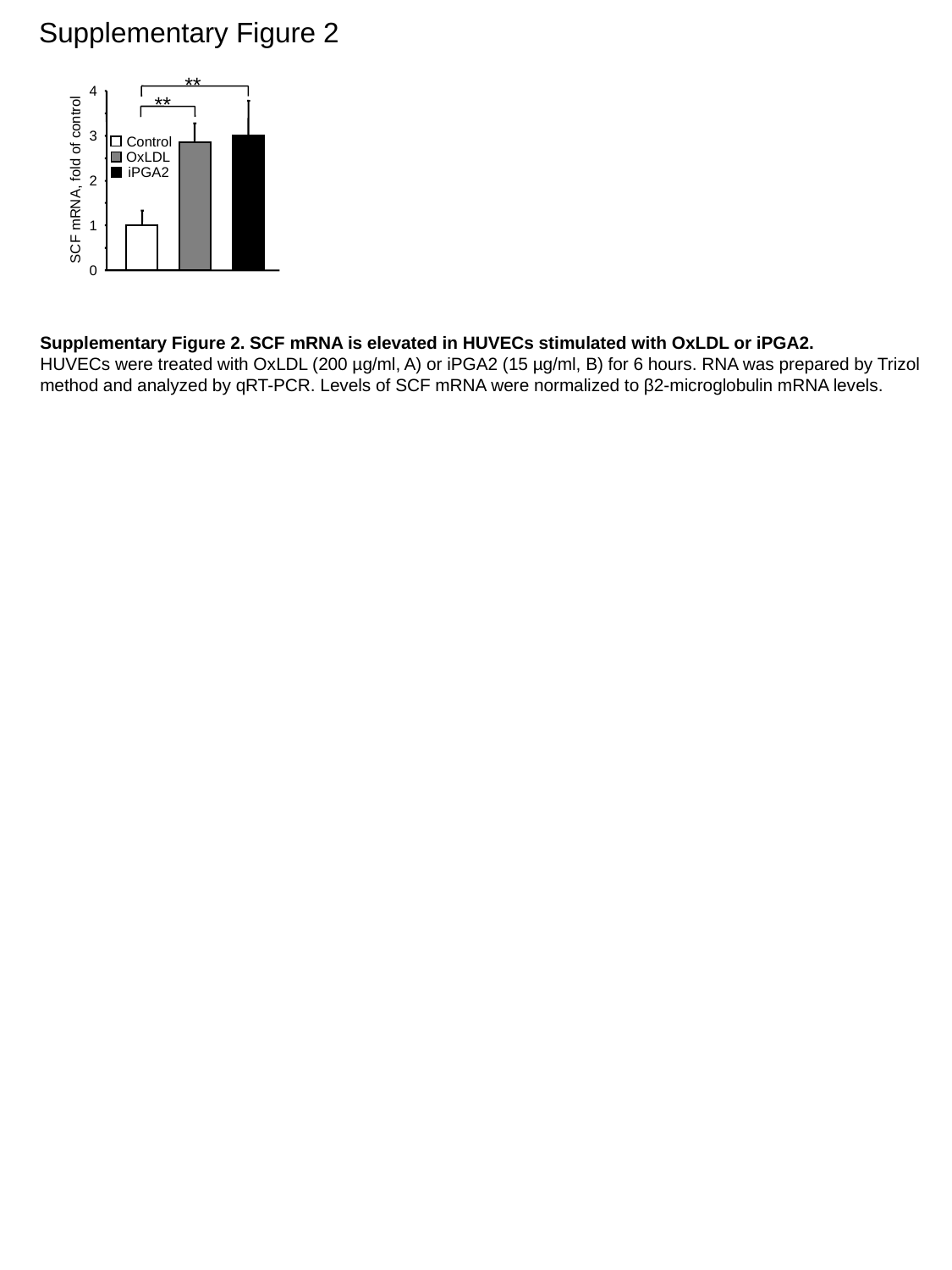

Supplementary Figure 2
**
4
**
Control
OxLDL
iPGA2
3
SCF mRNA, fold of control
2
1
0
Supplementary Figure 2. SCF mRNA is elevated in HUVECs stimulated with OxLDL or iPGA2.
HUVECs were treated with OxLDL (200 µg/ml, A) or iPGA2 (15 µg/ml, B) for 6 hours. RNA was prepared by Trizol method and analyzed by qRT-PCR. Levels of SCF mRNA were normalized to β2-microglobulin mRNA levels.

## Slide 4
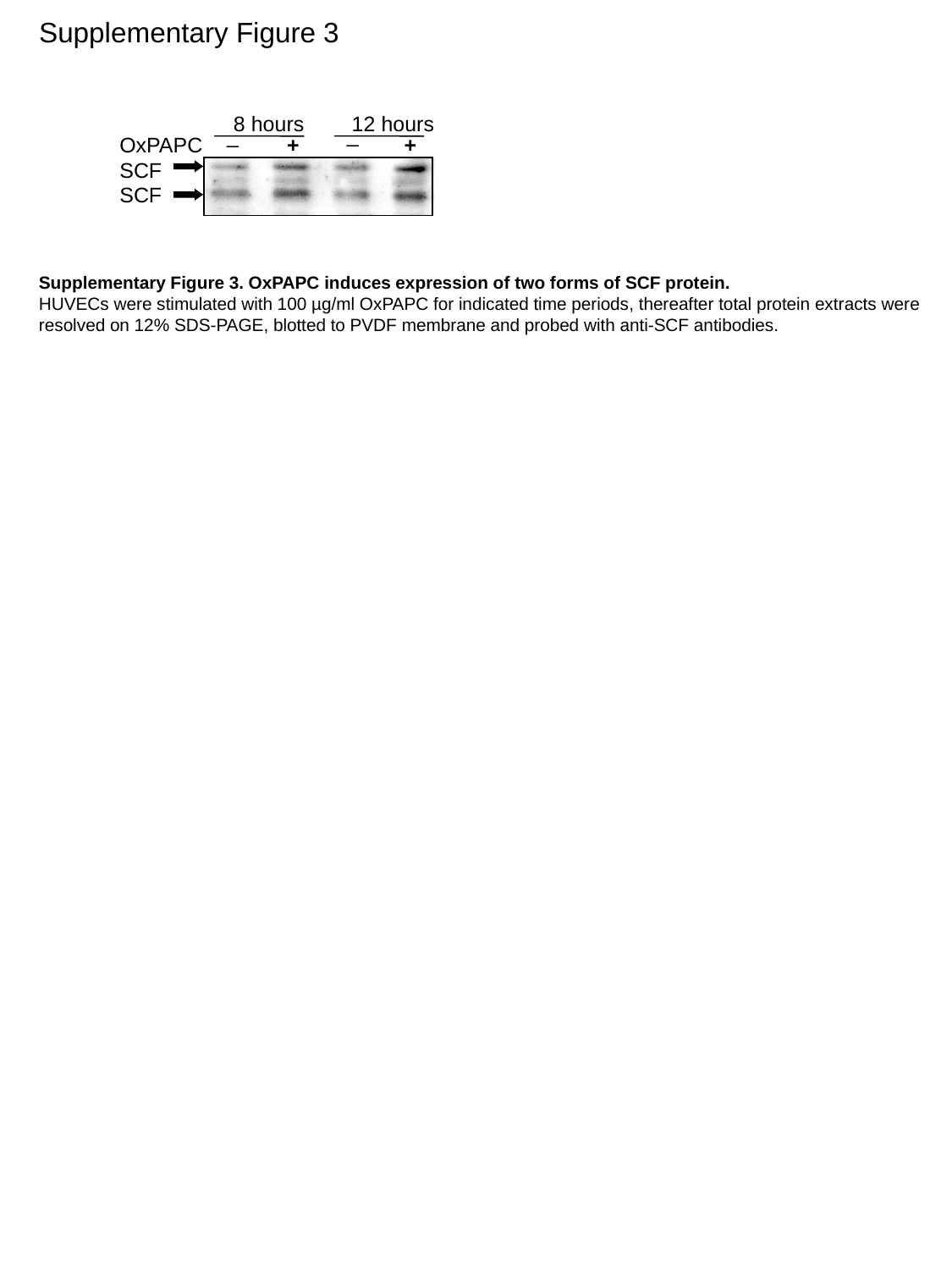

Supplementary Figure 3
8 hours
12 hours
_
_
OxPAPC
+
+
SCF
4
SCF
Supplementary Figure 3. OxPAPC induces expression of two forms of SCF protein.
HUVECs were stimulated with 100 µg/ml OxPAPC for indicated time periods, thereafter total protein extracts were resolved on 12% SDS-PAGE, blotted to PVDF membrane and probed with anti-SCF antibodies.

## Slide 5
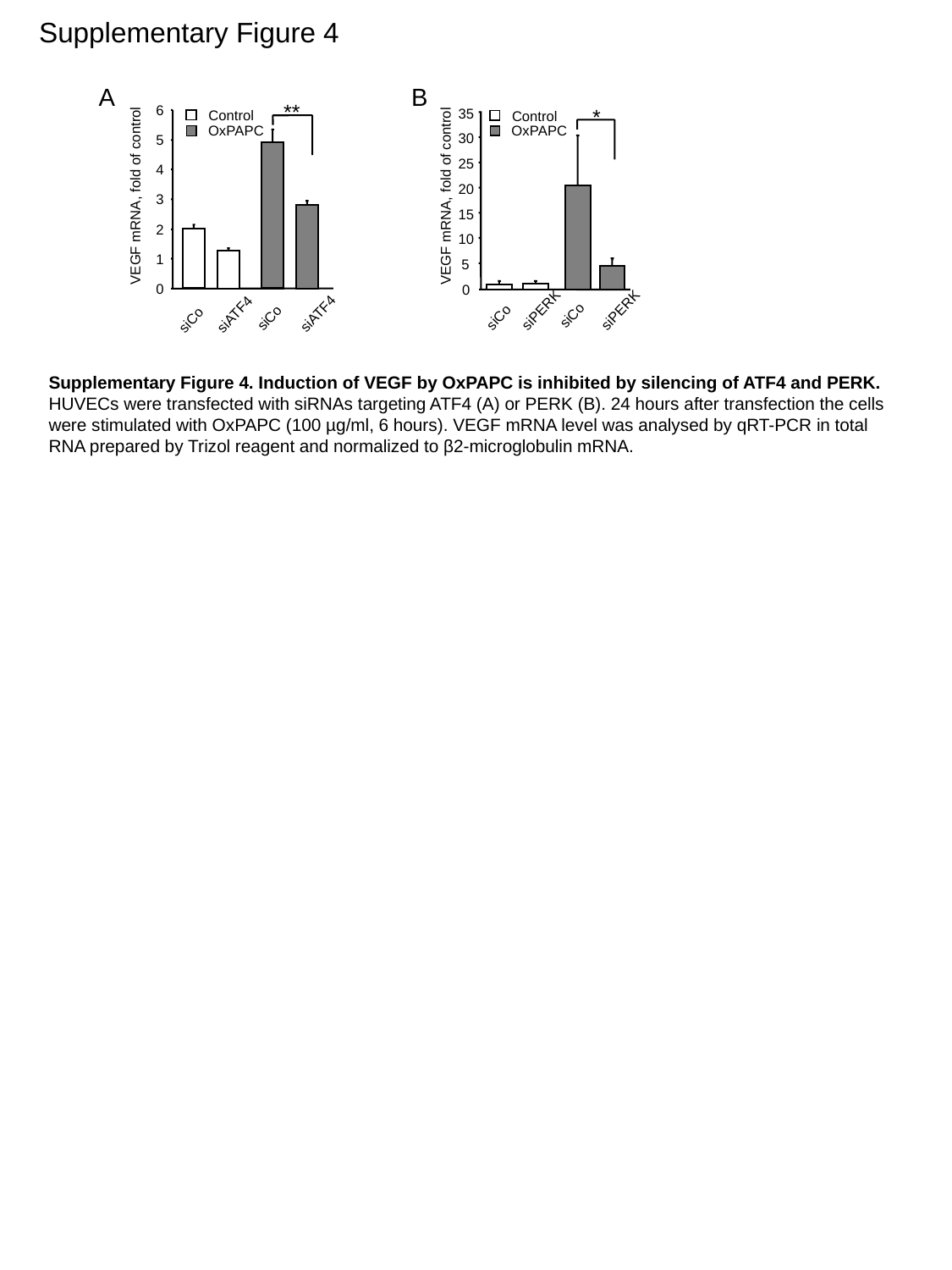

Supplementary Figure 4
A
B
**
*
Control
OxPAPC
Control
OxPAPC
6
35
30
5
25
4
20
VEGF mRNA, fold of control
VEGF mRNA, fold of control
3
15
2
10
1
5
0
0
siPERK
siPERK
siATF4
siATF4
siCo
siCo
siCo
siCo
Supplementary Figure 4. Induction of VEGF by OxPAPC is inhibited by silencing of ATF4 and PERK.
HUVECs were transfected with siRNAs targeting ATF4 (A) or PERK (B). 24 hours after transfection the cells were stimulated with OxPAPC (100 µg/ml, 6 hours). VEGF mRNA level was analysed by qRT-PCR in total RNA prepared by Trizol reagent and normalized to β2-microglobulin mRNA.

## Slide 6
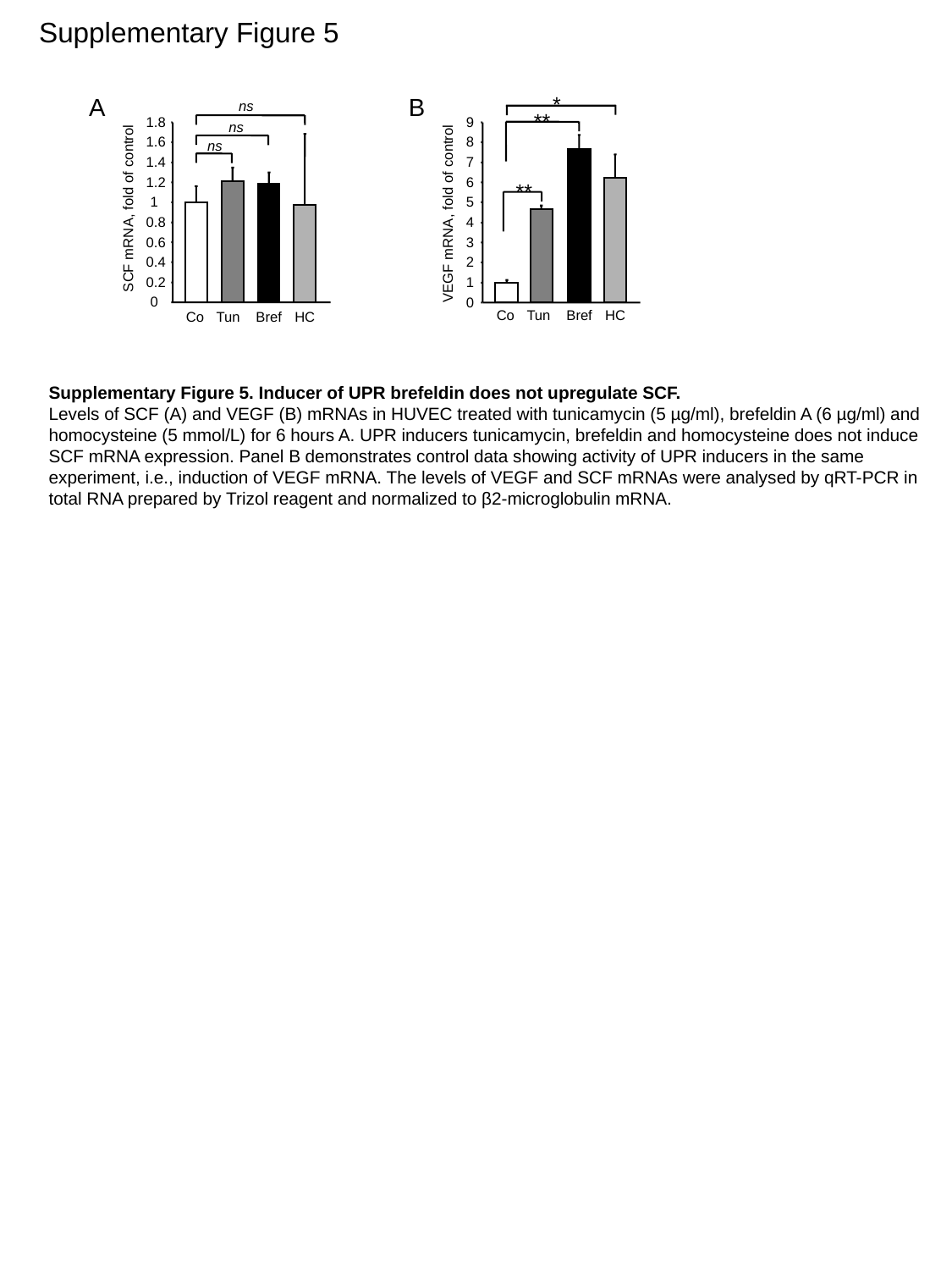

Supplementary Figure 5
A
B
*
ns
**
ns
1.8
9
8
7
6
5
4
3
2
1
0
Co
Tun
Bref
HC
ns
1.6
1.4
**
1.2
1
SCF mRNA, fold of control
VEGF mRNA, fold of control
0.8
0.6
0.4
0.2
0
Co
Tun
Bref
HC
Supplementary Figure 5. Inducer of UPR brefeldin does not upregulate SCF.
Levels of SCF (A) and VEGF (B) mRNAs in HUVEC treated with tunicamycin (5 µg/ml), brefeldin A (6 µg/ml) and homocysteine (5 mmol/L) for 6 hours A. UPR inducers tunicamycin, brefeldin and homocysteine does not induce SCF mRNA expression. Panel B demonstrates control data showing activity of UPR inducers in the same experiment, i.e., induction of VEGF mRNA. The levels of VEGF and SCF mRNAs were analysed by qRT-PCR in total RNA prepared by Trizol reagent and normalized to β2-microglobulin mRNA.

## Slide 7
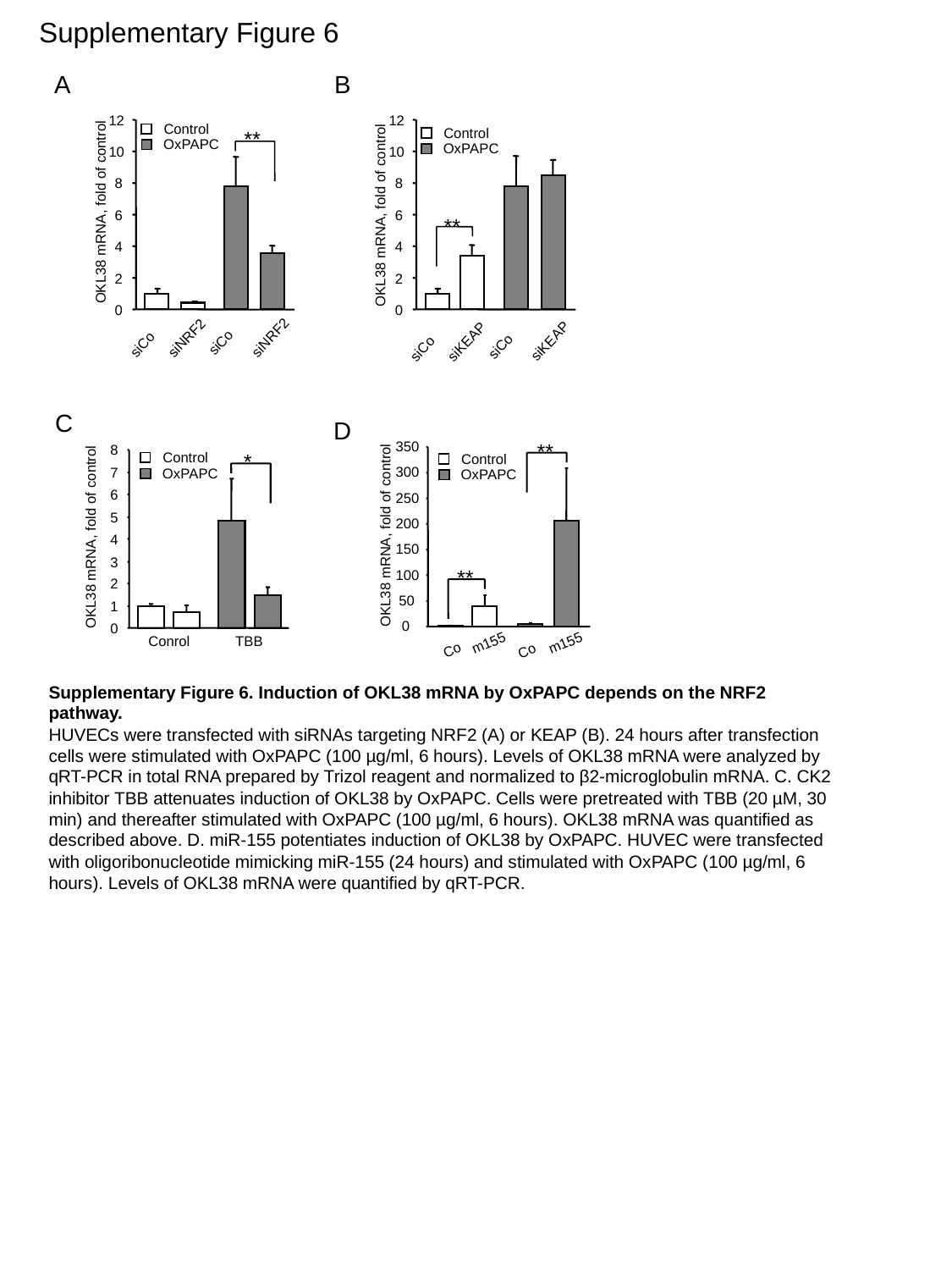

Supplementary Figure 6
A
B
12
12
Control
OxPAPC
Control
OxPAPC
**
10
10
8
8
OKL38 mRNA, fold of control
6
6
**
OKL38 mRNA, fold of control
4
4
2
2
0
0
siNRF2
siNRF2
siKEAP
siKEAP
siCo
siCo
siCo
siCo
C
D
**
350
Control
OxPAPC
300
250
200
OKL38 mRNA, fold of control
150
**
100
50
0
m155
m155
Co
Co
8
*
Control
OxPAPC
7
6
5
OKL38 mRNA, fold of control
4
3
2
1
0
Conrol
TBB
Supplementary Figure 6. Induction of OKL38 mRNA by OxPAPC depends on the NRF2 pathway.
HUVECs were transfected with siRNAs targeting NRF2 (A) or KEAP (B). 24 hours after transfection cells were stimulated with OxPAPC (100 µg/ml, 6 hours). Levels of OKL38 mRNA were analyzed by qRT-PCR in total RNA prepared by Trizol reagent and normalized to β2-microglobulin mRNA. C. CK2 inhibitor TBB attenuates induction of OKL38 by OxPAPC. Cells were pretreated with TBB (20 µM, 30 min) and thereafter stimulated with OxPAPC (100 µg/ml, 6 hours). OKL38 mRNA was quantified as described above. D. miR-155 potentiates induction of OKL38 by OxPAPC. HUVEC were transfected with oligoribonucleotide mimicking miR-155 (24 hours) and stimulated with OxPAPC (100 µg/ml, 6 hours). Levels of OKL38 mRNA were quantified by qRT-PCR.
